# Supplementary material for: A trimeric CrRLK1L-LLG1 complex genetically modulates SUMM2-mediated autoimmunity
Source: Nat Commun. 2020 Sep 25;11:4859. doi: 10.1038/s41467-020-18600-8 (PMC7519094; doi:10.1038/s41467-020-18600-8)
Supplement: Supplementary file 1 — Supplementary Information [file 41467_2020_18600_MOESM1_ESM.pdf]

Supplementary information

A trimeric CrRLK1L-LLG1 complex genetically modulates SUMM2-mediated autoimmunity

Yanyan Huang, Chuanchun Yin, Jun Liu, Baomin Feng, Dongdong Ge, Liang Kong, Fausto Andres Ortiz-Morea, Julia Richter, Marie-Theres Hauser, Wen-Ming Wang, Libo Shan, and Ping He

Contents

Supplementary Figure 1. Screen of *CrRLK1L* family members for *mekk1* suppressors.

Supplementary Figure 2. LETs do not regulate MPK4-mediated root development.

Supplementary Figure 3. LET2/MDS1 specifically promotes LET1 phosphorylation.

Supplementary Figure 4. The *llg1*, but not *lre*, *llg2*, nor *llg3* mutants suppress autoimmunity by silencing *MEKK1*.

Supplementary Figure 5. The *llg1* mutants regulate *mekk1* and *mpk4* cell death.

Supplementary Figure 6. Genotyping of *llg1-1mkk1/2* and *llg1-1mpk4* mutants.

Supplementary Figure 7. The *llg1-3* mutant suppresses the cell death triggered by MEKK2, not by SUMM2ac.

Supplementary Table 1. Primers used in this study.

a

NO.	T-DNA lines	AGI-Name	Predicted insertion site	Genotype	Cell death suppressor
1	*let2-1* (SALK_139579)	AT5G38990-*MDS1*	exon	HOMO	Yes
2	*let2-2 (*SALK_066322)		exon	HOMO	Yes
3	*mds3-1* (SALK_074670C)	AT5G39020-*MDS3*	exon	HOMO	No
4	*mds4-1* (SALK_007613C)	AT5G39030-*MDS4*	exon	HOMO	No
5	*fer-4* (CS69044)	AT3G51550-*FER*	exon	HOMO	No
6	SALK_029056C		exon	HOMO	No
7	*anx2-2* (SALK_133057C)	AT5G28680-*ANX2*	exon	HOMO	No
8	*anx1-1* (SALK_016179C)	AT3G04690-*ANX1*	exon	HOMO	No
9	*herk2* (SALK_105055C)	AT1G30570-*HERK2*	exon	HOMO	No
10	*cap1-1* (SALK_083442C)	AT5G61350-*ERULUS*	exon	HOMO	No
11	*herk1-1*(SALK008043C)<br>*the1-4*(CS829966)	AT3G46290-*HERK1*<br>AT5G54380-*THE1*	Double mutant	HOMO	No
12	*curvy1* (SALK_018797C)	AT2G39360-*CVY1*	exon	HOMO	No
13	*anj-1* (SALK_114667C)	AT5G59700-*ANJEA*	exon	HOMO	No
14	*herk1-1* (SALK_008043C)	AT3G46290-*HERK1*	exon	HOMO	No
15	*mds2-1* (SALK_007108)	AT5G39000-*MDS2*	exon	HOMO	No
16	SAIL_907_G02	AT5G24010	exon	WT	No
17	SAIL_809_D01		exon	WT	No
18	*bups1-T-1* (SALK_033062)	AT4G39110-*BUPS1*	exon	WT	No
19	*bups1-T-3* (SAIL_33_C06)		exon	WT	No
20	*bups2* (SAIL_448_D02)	AT2G21480-*BUPS2*	exon	WT	No

b

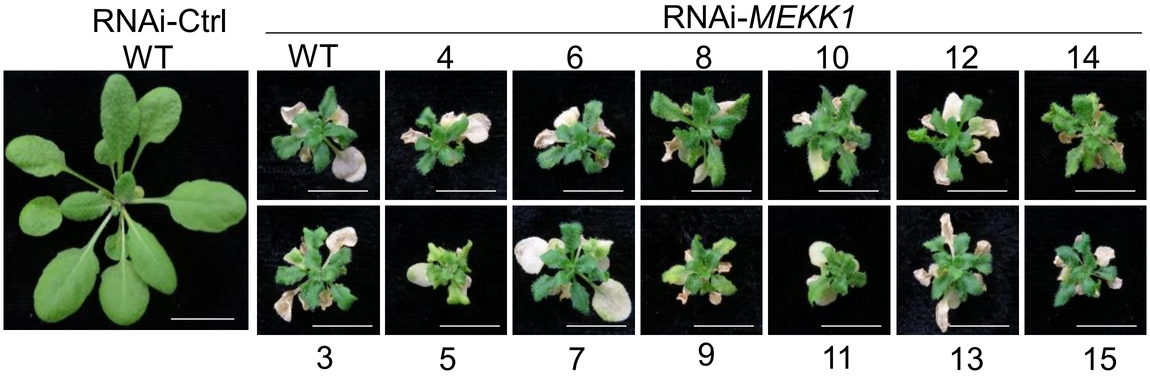

Supplementary Figure 1. Screen of *Cr*RLK1L family members for *mekk1* suppressors.

a. The detailed information of T-DNA insertion lines of *Cr*RLK1L family members in RNAi-*MEKK1* assays. HOMO indicates that the mutant is homozygous.

b. The phenotype induced by RNAi-*MEKK1*. The plant images were taken at three weeks after inoculation with Agrobacterium carrying RNAi-*MEKK1* vector. The number corresponds to the mutant marked with the same number in **(a)**. Scale bar, 1cm.

The above experiments were repeated twice with similar results.

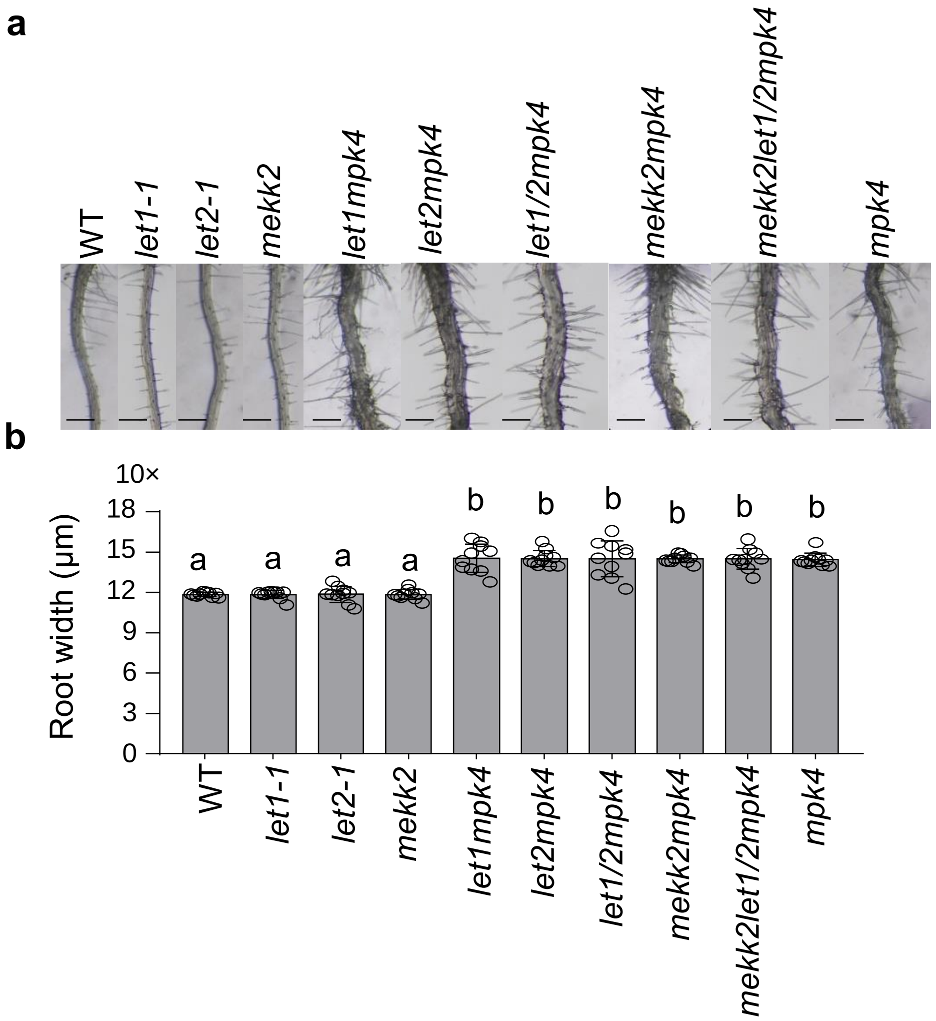

Supplementary Figure 2. LETs do not regulate MPK4-mediated root development.

a. The *let* mutants do not affect the root width in the *mpk4* mutant. The root images were taken at six days after germination on a ½MS plate under a stereoscopy. Scale bar, 200 μm.

b. Quantification of root width of plants in **(a)**. The root width of the indicated lines was measured by image J. The data are shown as the mean ± SE (*n*=10). $P = 4.4 \times 10^{-10}$ (column 1 and 5), $P = 4.42 \times 10^{-10}$ (column 1 and 6), $P = 4.48 \times 10^{-10}$ (column 1 and 7), $P = 4.50 \times 10^{-10}$ (column 1 and 8), $P = 4.50 \times 10^{-10}$ (column 1 and 9), $P = 4.67 \times 10^{-10}$ (column 1 and 10). The different letters indicate the significant difference determined by one-way ANOVA followed by the Tukey test ($P < 0.05$).

The above experiments were repeated three times with similar results.

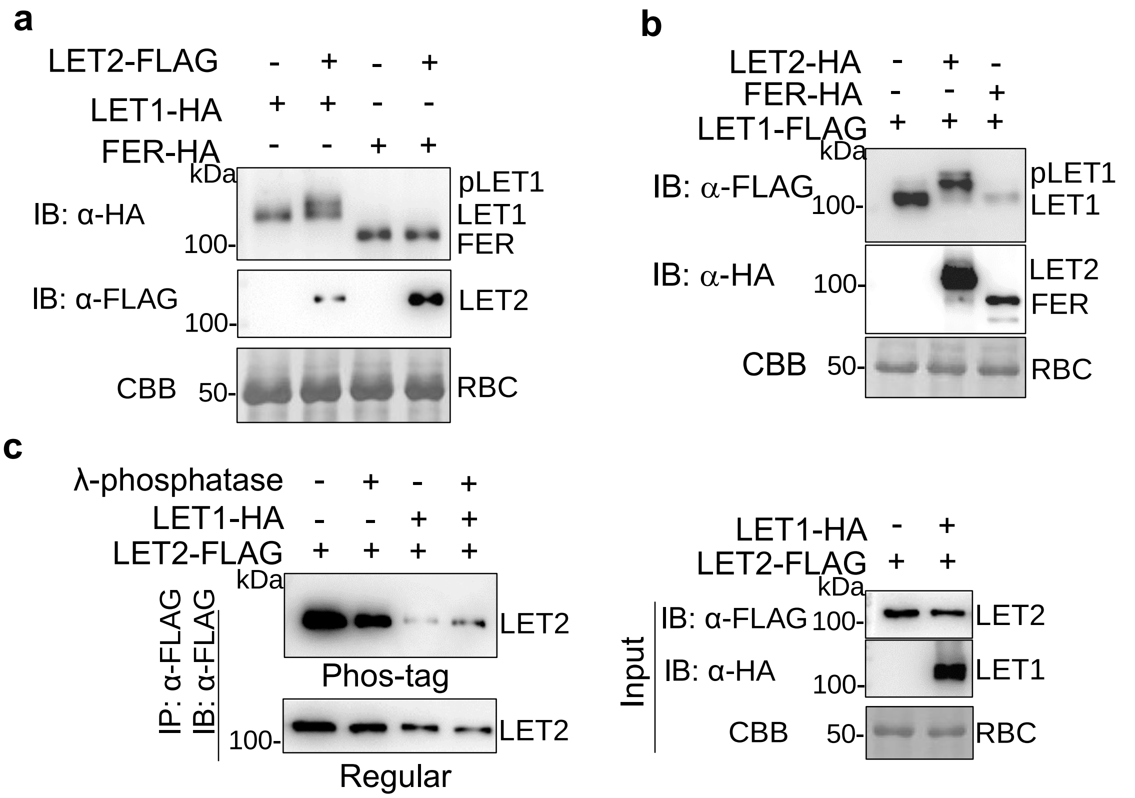

Supplementary Figure 3. LET2/MDS1 specifically promotes LET1 phosphorylation.
a. LET2/MDS1 promotes LET1, not FER, phosphorylation. LET1-HA or FER-HA was co-expressed with Ctrl or LET2-FLAG in protoplasts for 12 hr. The proteins were separated by 10% SDS-PAGE and detected by an α-FLAG or α-HA antibody. CBB staining of RBC was used as a loading control.
b. LET2/MDS1, not FER, promotes LET1 phosphorylation. LET2-HA or FER-HA was co-expressed with LET1-FLAG in protoplasts for 12 hr. The mobility-shift of LET1-FLAG was detected with a 7.5% SDS-PAGE. CBB staining of RBC was used as a loading control.
c. LET1 does not promote LET2/MDS1 phosphorylation. LET2-FLAG was co-expressed with Ctrl or LET1-HA in protoplasts. LET2-FLAG proteins were immunoprecipitated with α-FLAG affinity beads, subsequently treated without or with 0.5 μL (200 U) of λ-phosphatase for 1 hr. The proteins were separated by either Phos-tag SDS-PAGE (top left) or regular SDS-PAGE (bottom left). The proteins before immunoprecipitation were immunoblotted by an α-HA or α-FLAG antibody as inputs (right). CBB staining of RBC was used as a loading control.
The above experiments were repeated three times with similar results.

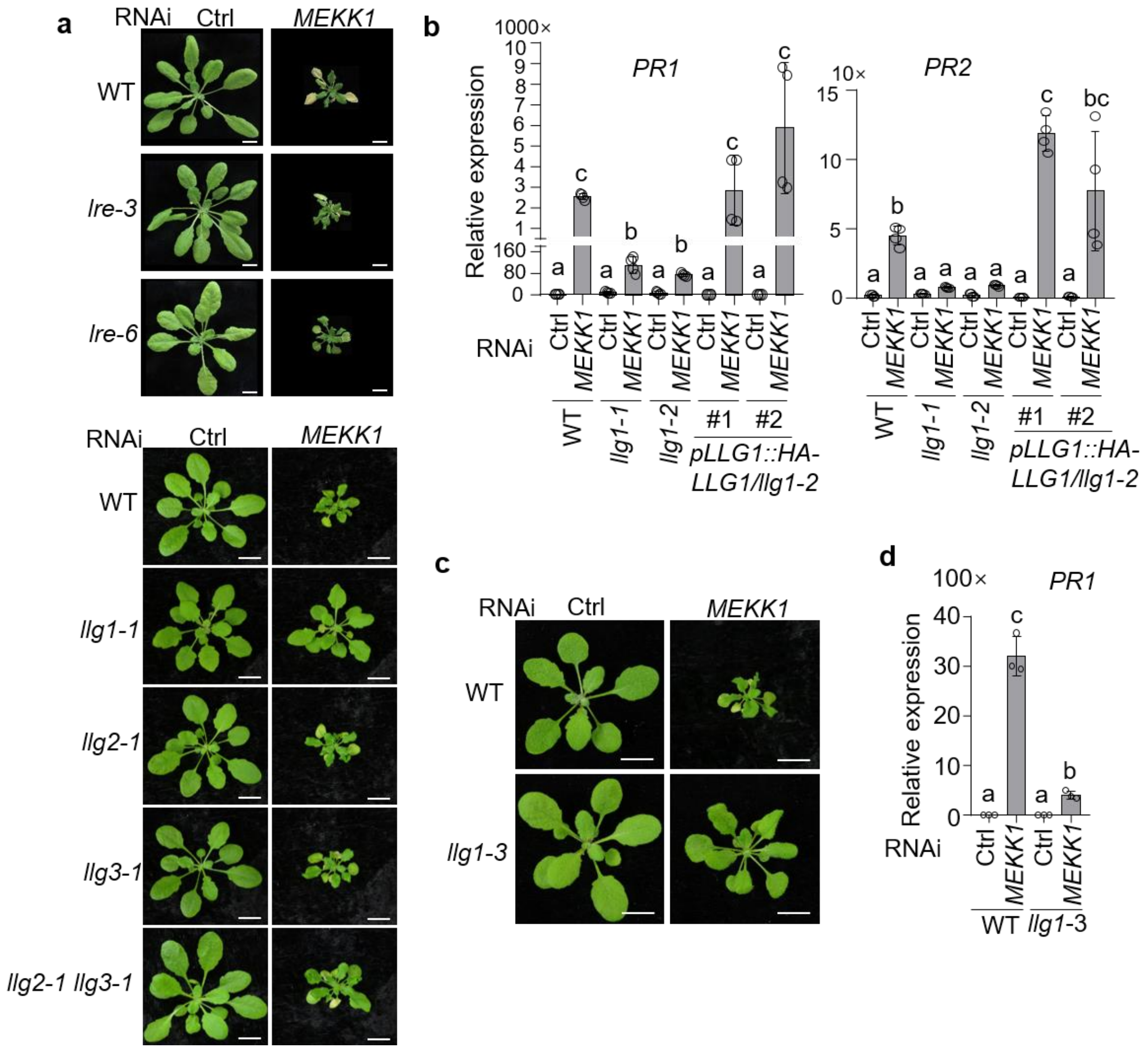

Supplementary Figure 4. The *llg1*, but not *lre, llg2,* nor *llg3* mutants suppress autoimmunity by silencing *MEKK1*.

a. The *lre, llg2, llg3* mutants do not suppress the cell death induced by silencing *MEKK1*. The images were taken at three weeks after inoculation of Agrobacterium carrying the indicate VIGS vector. Scale bar, 0.5 cm (top for *lre* mutants), 1 cm (bottom for *llg2* and *llg3* mutants).

b. The *llg1* mutants suppress the expression of *PR* genes induced by silencing *MEKK1*. The expression of *PR1* (left) and *PR2* (right) was normalized to the expression of *UBQ10* and the data are shown as the means ± SE of four biological repeats (n=4). P =1.20 × 10^{-2} (*PR1*, column 2 and 4), P =0.99 × 10^{-2} (*PR1*, column 2 and 6), P =2.83× 10^{-2} (*PR2*, column 2 and 4), P =4.03 × 10^{-2} (*PR2*, column 2 and 6). The different letters indicate the significant difference determined by one-way ANOVA followed by the Tukey test (P < 0.05).

c. The *llg1-3* mutant partially suppresses the cell death induced by silencing *MEKK1*. The assay was done as in **(a)**. Scale bar, 1 cm.

d. The expression of *PR1* induced by silencing *MEKK1* is suppressed in *llg1-3*. The expression of *PR1* was normalized to the expression of *UBQ10* and the data are shown

as the means ± SE of three biological repeats (n=3). P =8.23× 10^{-7} (column 2 and 4). The different letters indicate the significant difference determined by one-way ANOVA followed by the Tukey test ($P < 0.05$).

The above experiments were repeated three times with similar results.

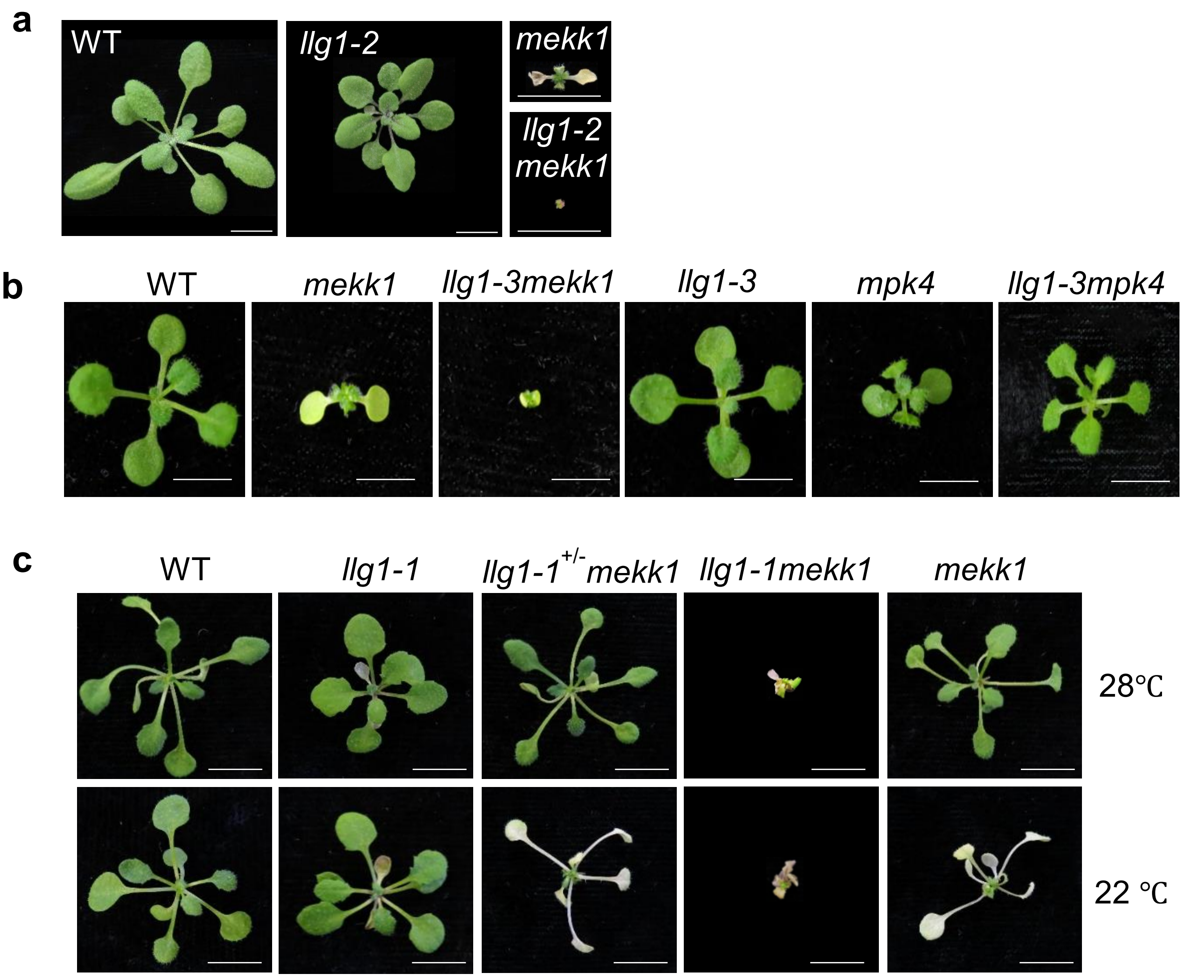

Supplementary Figure 5. The *llg1* mutants regulate *mekk1* and *mpk4* cell death.
a. The *llg1-2* mutant enhances the growth defects of *mekk1*. The images were paragraphed at three weeks after germination with plants grown on soil. Scale bar, 1cm.
b. The *llg1-3* mutant enhances *mekk1,* but suppresses *mpk4* growth defects. Plants grown on ½MS plates were photographed at two weeks after germination. Scale bar, 1cm.
c. The growth defects of *llg1-1mekk1* mutant do not recover at 28℃. The indicated plants were first grown in a growth room at 22℃ for 3 days after germination and then moved to a 28℃ growth room for three weeks (upper panel). Finally, the plants were moved back to a 22℃ growth room for another three days (low panel). Scale bar, 1cm.
The above experiments were repeated three times with similar results.

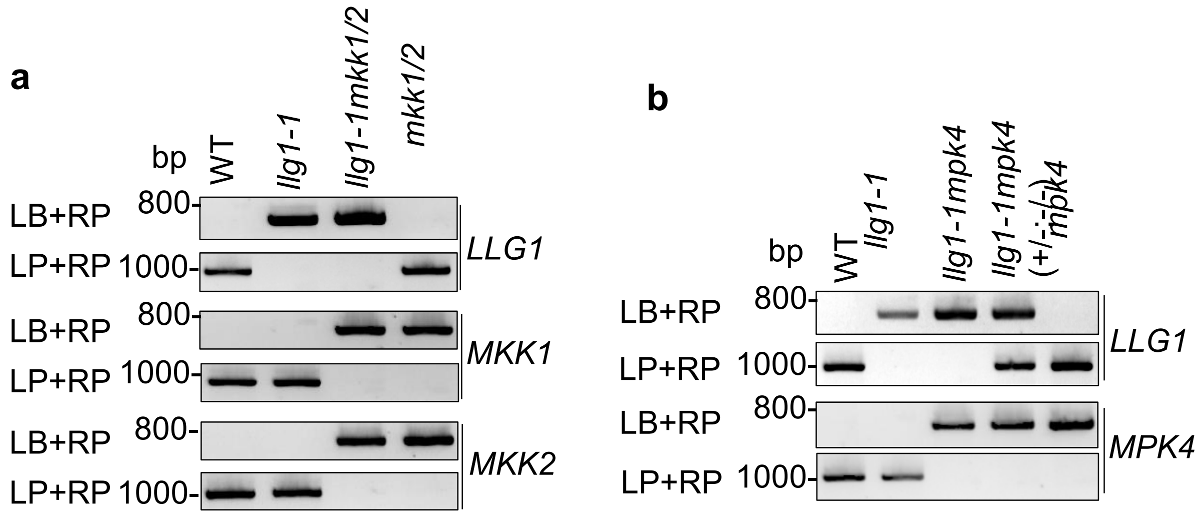

Supplementary Figure 6. Genotyping of *llg1-1mkk1/2* and *llg1-1mpk4* mutants.
WT, *llg1-1, mkk1/2* and *mpk4* were used for control for genotyping of *llg1-1mkk1/2* (**a**) and *llg1-1mpk4* (**b**) mutants. The primers used for genotyping are listed in Supplementary Table 1. The primer pair of LP and RP amplified genomic DNA, and the primer pair of LB and RP amplified T-DNA insertion.
The above experiments were repeated twice with similar results.

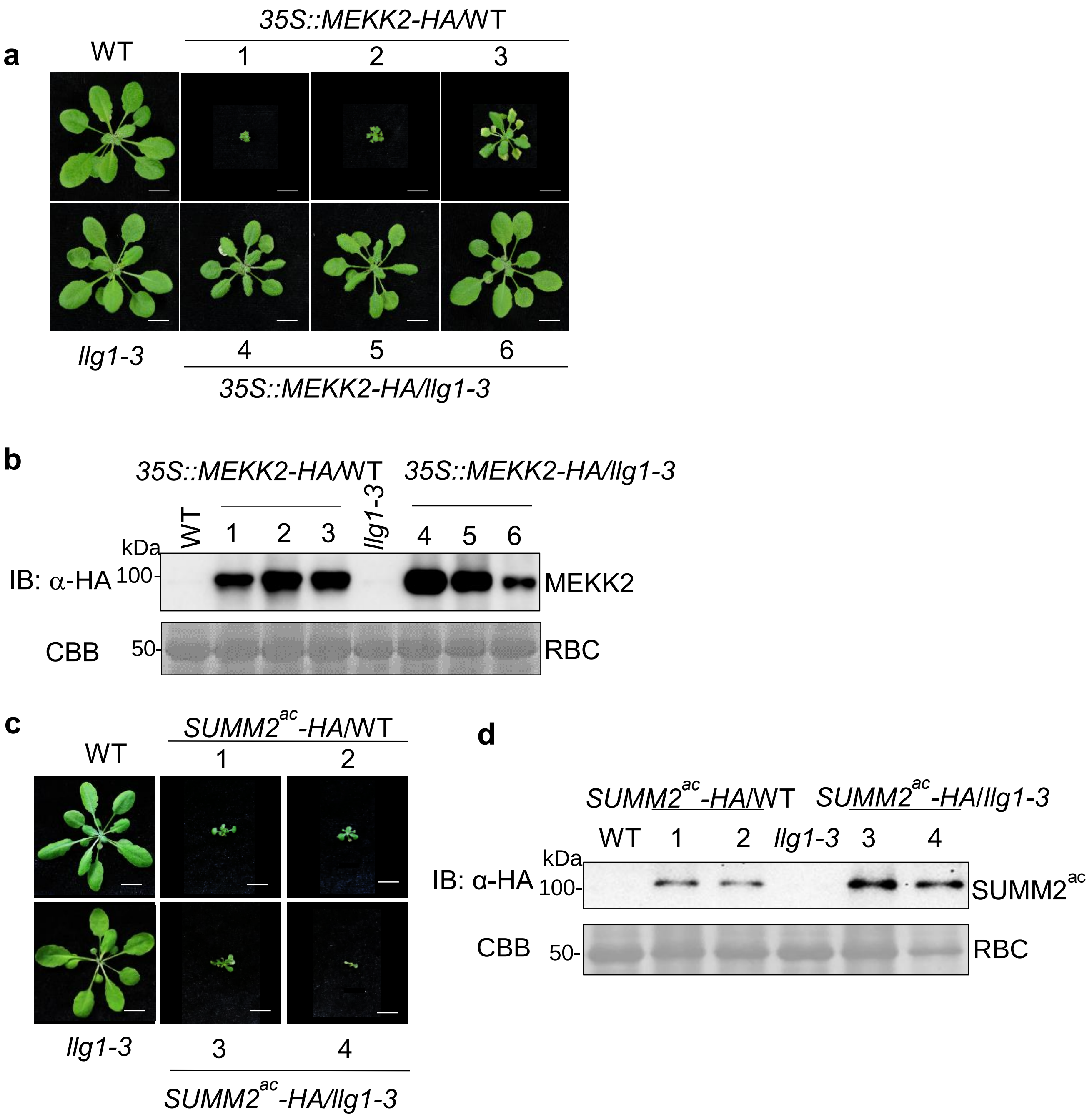

Supplementary Figure 7. The *llg1-3* mutant suppresses the cell death triggered by MEKK2, not by SUMM2^{ac}.

a. The *llg1-3* mutant largely suppresses the cell death triggered by overexpressing *MEKK2*. The images were photographed for T_1 generation of transgenic lines overexpressing *MEKK2-HA* in WT and *llg1-3* at four weeks after germination. Scale bar, 1cm.

b. The protein accumulation in transgenic lines overexpressing *MEKK2-HA*. Total proteins were isolated from plants in **(a)** and immunoblotted using an α-HA antibody (top panel). CBB staining of RBC is shown as the loading control (bottom panel).

c. The growth defects triggered by overexpressing *SUMM2*^{ac}*-HA* are similar in WT and *llg1-3*. Two representative plants in each background were photographed at four weeks after germination. Scale bar, 1 cm.
d. Protein expression of SUMM2^{ac}-HA in transgenic plants in **(c)**. The assay was performed as in **(b)**.
The above experiments were repeated twice with similar results.

Supplementary Table 1. Primers used in this study

Genotyping primers

Name	Sequence
SALK_127359 -LP	TGAGTCAAGGCTTGACATGTG
SALK_127359-RP	TTTAAGCAATGGATGGTCGAG
SALK_045687-LP	AAACGAATTAATCCCGGTTTG
SALK_045687-RP	CAAGGACTCAACGAATTCGAG
SALK_007613-LP	GACTTGCATCCTCTGGTGAAG
SALK_007613-RP	TCCTTCCATCATTTCAACGAC
SALK_029056-LP	TGGTAGGATTCCGTTAAAATGC
SALK_029056-RP	CAGAGTATTTCAGACGGCAGC
SALK_018797-LP	TTGGTGGTGCATTAGGAAAAG
SALK_018797-RP	ACAACAAATCTCCCATTGCTG
SALK_074670-LP	GCAACATCATAAAGAACAACCC
SALK_074670-RP	TAGCATACACATCATACGGCG
SALK_105055-LP	ACTGGTCACAATGCTACTGCC
SALK_105055-RP	CTTACCAAACCCTCCAACTCC
SALK_008043-LP	ATGTGACTTGGGAGTTCGATG
SALK_008043-RP	TGCAGATTTCACGTCTCTGTG
SALK_083442-LP	TTTATCAACGCCGTTGAAATC
SALK_083442-RP	ATTTTGTGTCGCGGTCTGTAG
SALK_114667-RP	GCACCACTCAAAGTGTTGGA
SALK_114667-LP	TGGATCATCAACCAACGTCA
SAIL_907_G02-LP	ACCTGGCTCGAGTTTTCTCTC
SAIL_907_G02-RP	AACACAAAACCTCCCAAAACC
SALK_033062-LP	CTGAGCTCCAAGTCCAGATTG
SALK_033062-RP	ACACTTCTCTGCAGCTTCAGC
SAIL_33-C06-LP	TTTCCATACAAGTGGTCCCTG
SAIL_33-C06-RP	GATCTCTGATTCTGGCACTGC
SAIL_448_D02-LP	TGCTTCATGCTCTCAAAGATC
SAIL_448_D02-RP	CTCTGCTCCTGTTGCGTAAAC
SALK_139579-LP	TCAATGGACGTAACTTTGAGG
SALK_139579-RP	AGTGAAACGGTTGACGTTGAC
SALK_206468-LP	AACGCCACTAAAAGGAAAAGG
SALK_206468-RP	TCACCACTTGGCATTAGATCC
SAIL_809_D01-LP	CGAGAGAGAGACCGACATTTG
SAIL_809_D01-RP	CAACAGCTCACGGATAAGTCC
SALK_007108_LP	GGCGAAAAATGTTAACACCAC
SALK_007108_RP	CTTTTCAGGAGGGACAAAACC
fer-4_LP	AGATCACAGAGGGACGATTC
fer-4_RP	GCACCAAACACACAAAACCC
GAB1(R)	GTGGATTGATGTGATATCTCC

SAIL_103_E02_LP	TTGTATGGGTTGCAGGAAAAG
SAIL_103_E02_RP	TTCCTCCTCTCTGGTTTCTCC
SAIL_1234_C03-LP	TTGATGATGCTATGGAGCTCC
SAIL_1234_C03-RP	CAAAATCTTCTTTGCAGGCTG
SALK_118763.3-LP	CAAGCAAAGTCCTTGAAGGC
SALK_118763.3-RP	ACGGTTCATGTCTCCGAATC
SAIL_47_G04-LP	TCCGAGTGAGGAACAAACATC
SAIL_47_G04-RP	TGCCAAGAACTCACATTTTCC
SALK_040289_LP	GTCATTGGCAGAAGAGCAAAC
SALK_040289_RP	TGACGTAACGTCGGAACTAGG
CS66103-LP	CGCACACATGCACCTAAGTAG
CS66103-RP	TCCGACGTTACGTCATAATCC
SALK_150039-LP	CGCCGGACTAGTCTTATCTCC
SALK_150039-RP	TACATTTTTGCAGCCACTTTG
AT2g23200-genotypingF	CTCTTTAGCTCTCATCAATGCCA
AT2g23200-genotypingR	TGTTCCTCGCTGAATCTTTTC
mpk4-LP	TTGCTCTGAATACACAGCAGC
mpk4-RP	GTCTTAGAGATCAGCGGGGAC
mkk1-LP	ACGACCATTTCGTCTTCGTC
mkk1-RP	GGACATTGCGAGCTCAAGTT
FISH1-LP	CTGGGAATGGCGAAATCAAGGCATC
SAIL_511_H01-LP	TTCTTTTCCCAAATGGATTCC
SAIL_511_H01-RP	GTTAAAGCCATCCCTGACTCC
LBb1.3	ATTTTGCCGATTTCGGAAC
LB1	GCCTTTTCAGAAATGGATAAATAGCCTTGCTTCC

Cloning primers

Name	Sequence
NcoI(ATG)-BglII-(ATG)At5g38900-F	CATGCCATGGAGATCTATGATCTGTCACGTTTTAGTAATTT
SnaBI-SmaI-At5g38900-R	CATTACGTACCCGGGCCGTGCTTTAGGTTCATTGA
LET2-Km-F	CACTTGTTGCGGTTGAACGGCTGGAAATTA
LET2-Km-R	TAATTTCCAGCCGTTCAACCGCAACAAGTG
BamHI-At2g23200-F	CGGGATCCATGGAGAATTTCTGTTTTCAAGAC
StuI-At2g23200-R	GAAGGCCTTCTTGCATCAGAGATCTTCAACT
At2g23200-no BamHI-F	CGGTTACTTGGACCCAGAATATCTCC
At2g23200-no BamHI-R	GGAGATATTCTGGGTCCAAGTAACCG
LET1-K516E-F	ACCAAAGCCGCTATCGAACGAGGCAAAACC
LET1-K516E-R	GGTTTTGCCTCGTTCGATAGCGGCTTTGGT
BamHI-MPK4-F	GCCGGATCCATGTCGGCGGAGAGTTGTTT C
StuI-MPK4-R	GAAGGCCTCACTGAGTCTTGAGGATTGAAC
BamHI-MEKK2-F	CGGGATCCATGAAGAAGTCGTCGGATAA
StuI-MEKK2-R	GAAGGCCTTCTACGGATTAGCGGAGATG
BamHI-SUMM2-F	CGGGATCCATGGGAGCTTGTTTAACACTCTCG
StuI-SUMM2-R	GAAGGCCTCCGCACATAACTAACTTGCCATTC
SUMM2D478V-F	GTTAAAATGCATGTTGTGG TTCGG

SUMM2D478V-R	CCGAACCACAACATGCATTTTAAC
BamHI-LET1-ECD-F	GGAGGATCCATGGAGAATTTCTGTTTTCAAG
BamHI-LET1-exJM-F	GGAGGATCCATGGAGCAGCCTAGGTTGGCG
StuI-LET1-ECD-R	GGCAGGCCTAACCCGGGAACTGCTTCTAT
BglII-LLG1-F	TACAGATCTTTCATTTCAGATGGGGTCTTC
PstI-LLG1-R	CAGCTGCAGTCAGAACAACTTAACAAAAAC
BamHI-LET2ex-F	AGAGAACAGATTGGTGGATCCATGTCGTATGAGCCC ACTGATGT
HindIII-LET2ex-R	CTCGAGTGCGGCCGCAAGCTTTCATCTTGCATCAGA GATCTTCAACTGC
BamHI-LET2CD-F	AGAGAACAGATTGGTGGATCCATGAAGAGAAAGAAG AAGAGCAACG
HindIII-LET2CD-R	CTCGAGTGCGGCCGCAAGCTTTCACCGTGCTTTAGG TTCATTGATCT

qRT-PCR primers

Name	Sequence
UBQ10-qRT-F	AGATCCAGGACAAGGAAGGTATTC
UBQ10-qRT-R	CGCAGGACCAAGTGAAGAGTAG
PR1-F	GGTTAGCGAGAAGGCTAACTAC
PR1-R	CATCCGAGTCTCACTGACTTTC
PR2-F	GCATTCGCTGGATGTTTTG
PR2-R	CTTCAACCACACCAGCTTGGAC
