# Supplementary material for: A trimeric CrRLK1L-LLG1 complex genetically modulates SUMM2-mediated autoimmunity
Source: Nat Commun. 2020 Sep 25;11:4859. doi: 10.1038/s41467-020-18600-8 (PMC7519094; doi:10.1038/s41467-020-18600-8)
Supplement: Supplementary file 4 — Source Data [file 41467_2020_18600_MOESM4_ESM.zip › 246592_2_related_ms_4874231_qfn60y.pdf]

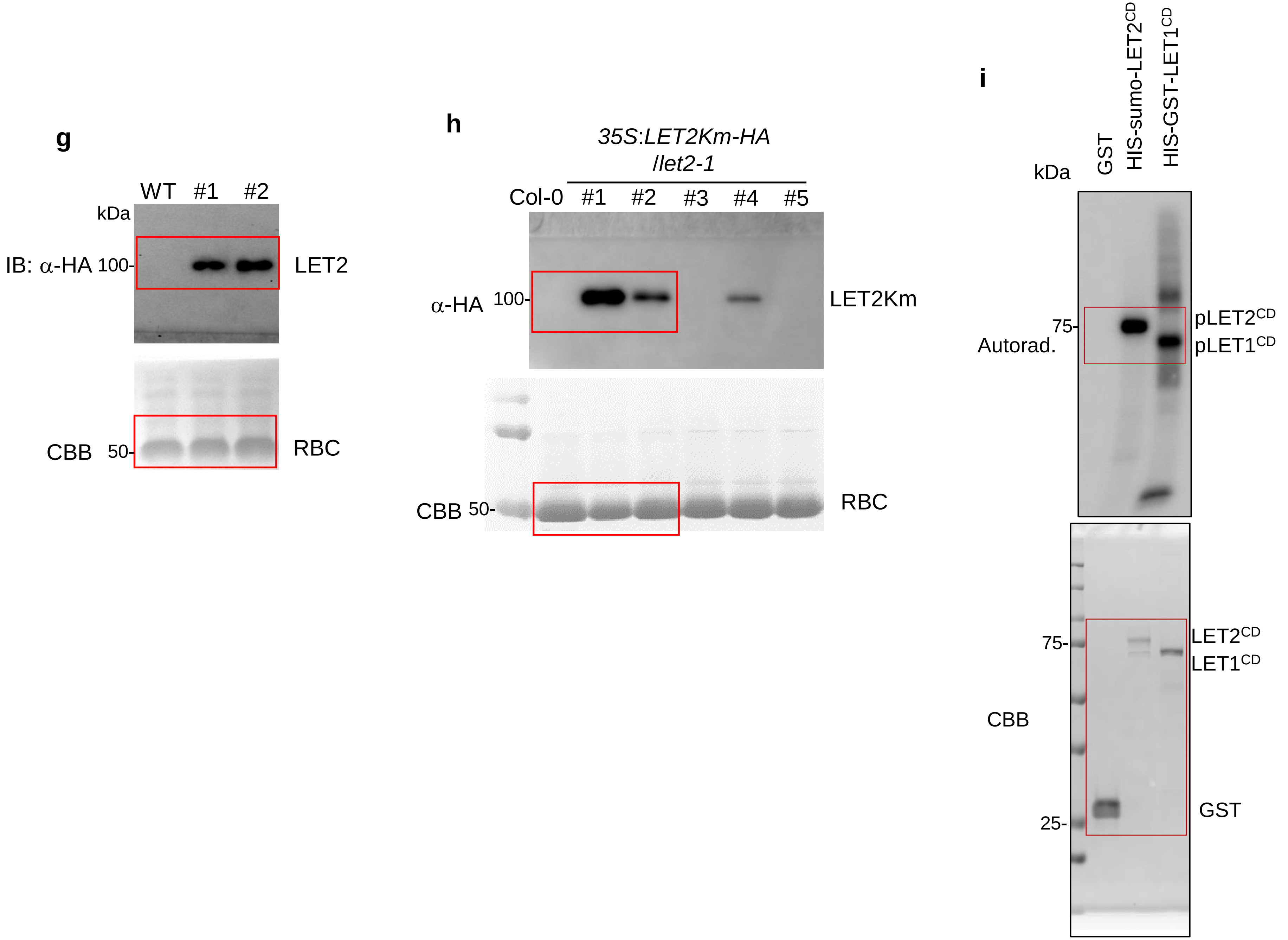

Source data Figure 1

b

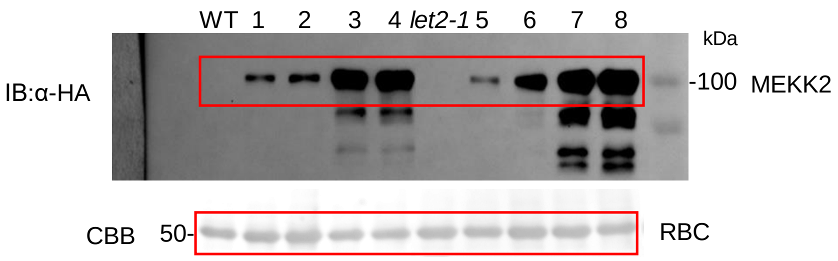

f

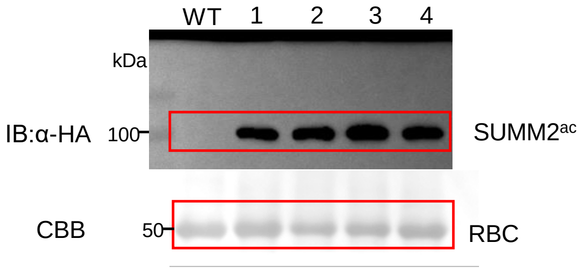

Source data Figure 3

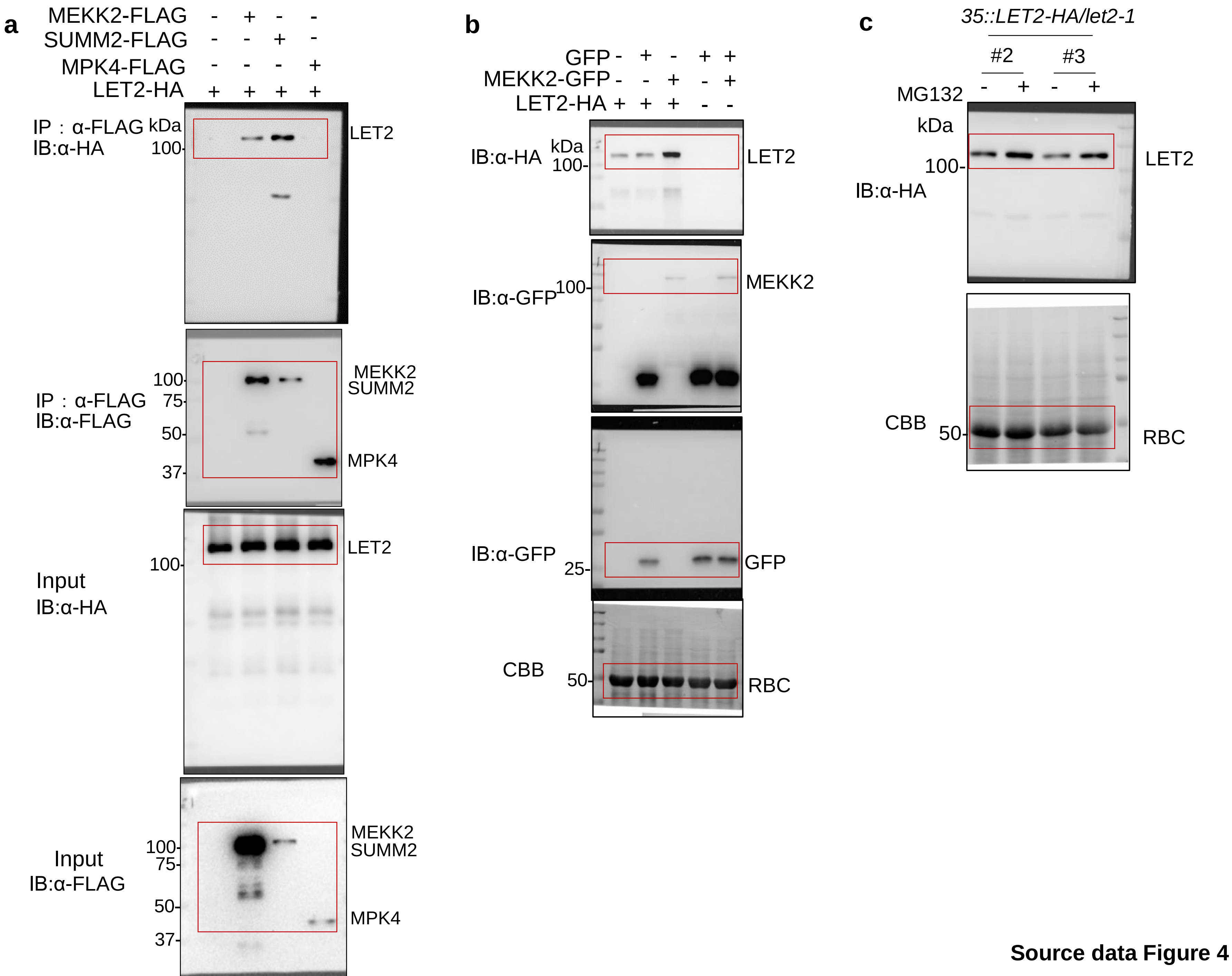

Source data Figure 4

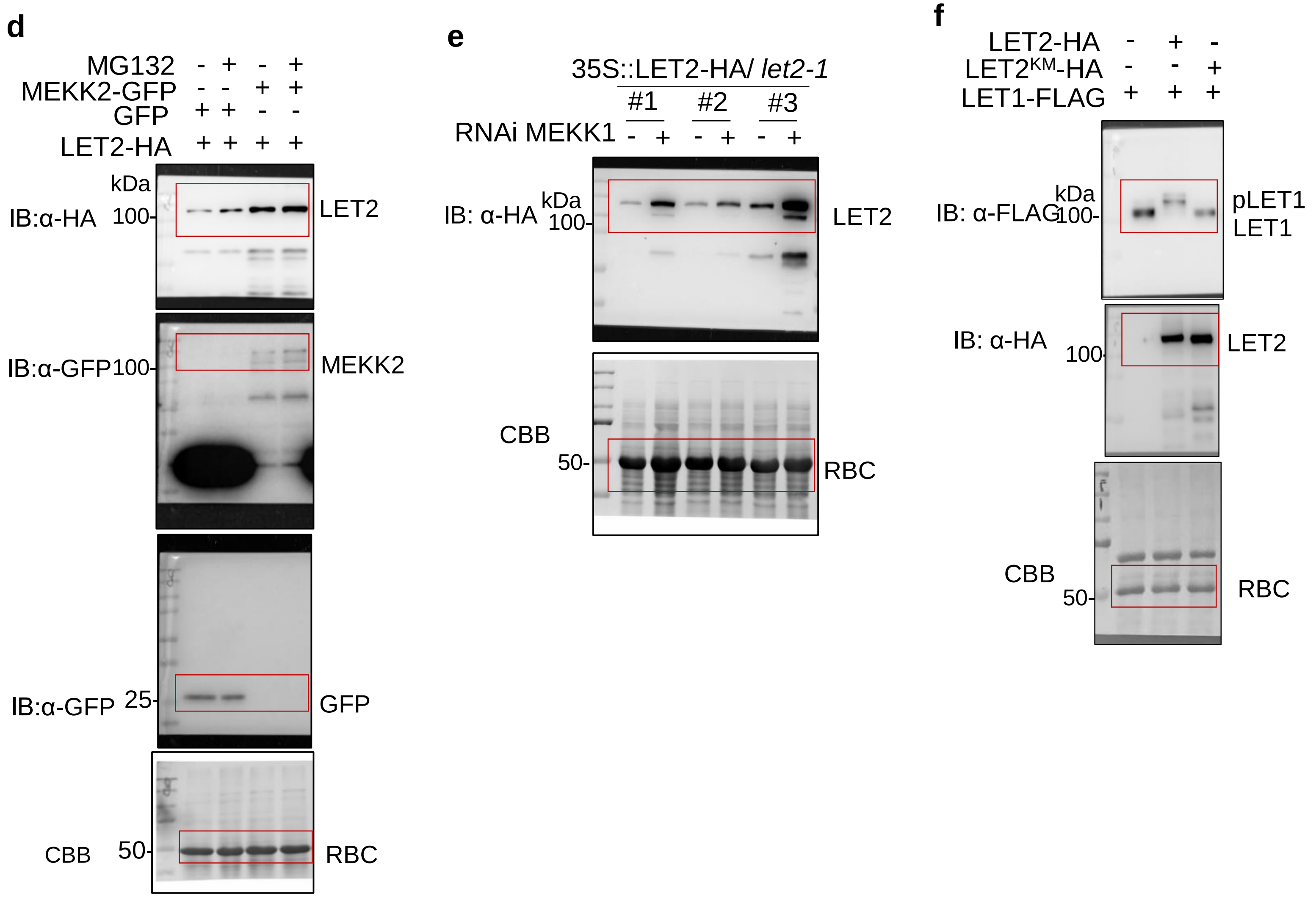

Source data Figure 4

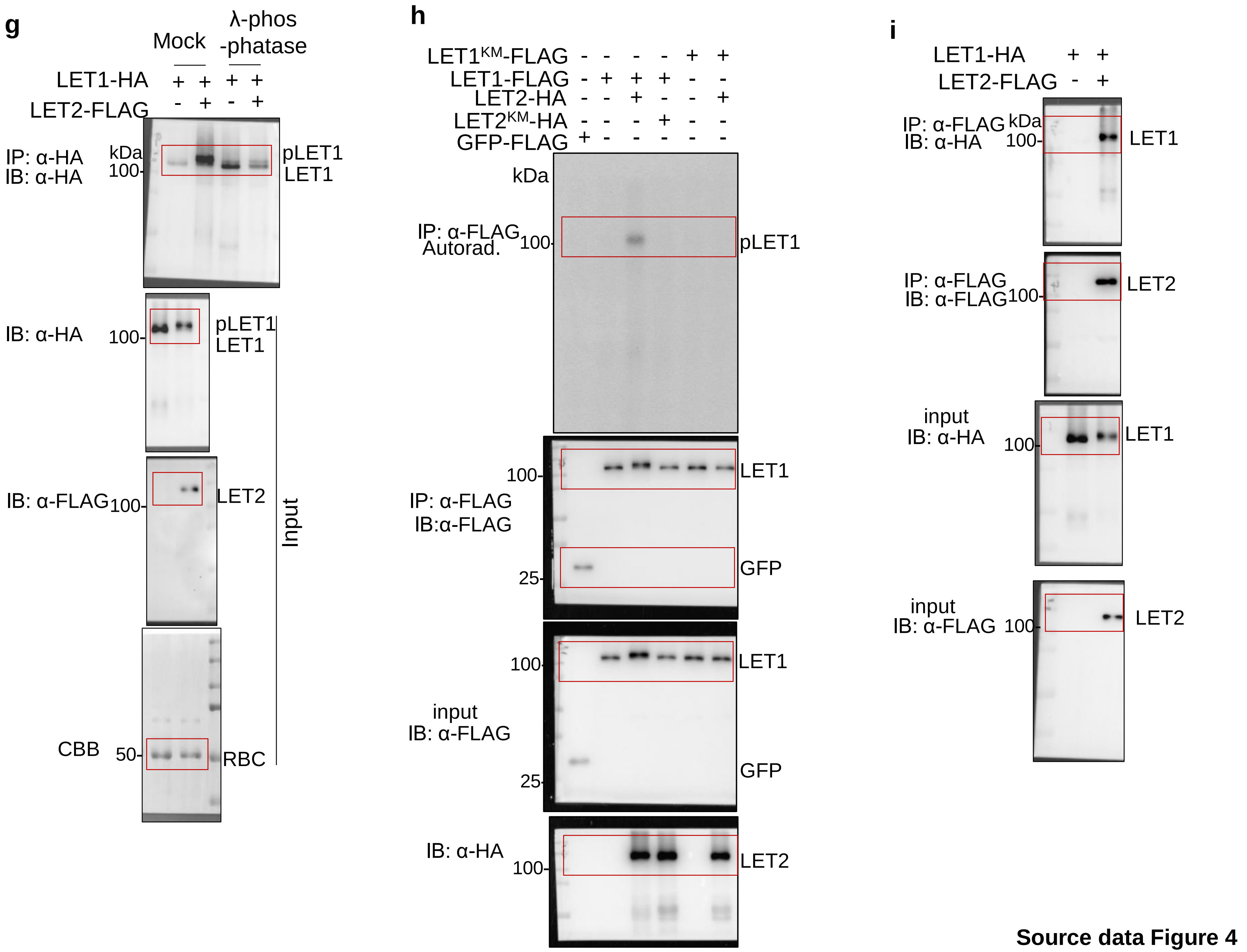

Source data Figure 4

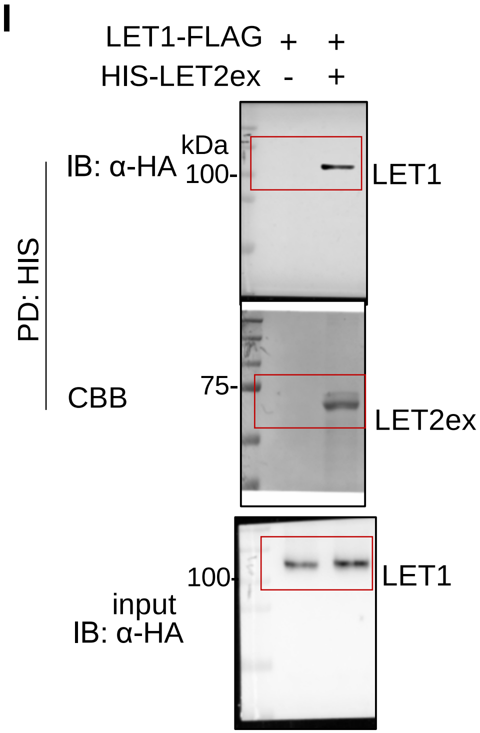

Source data Figure 4

d

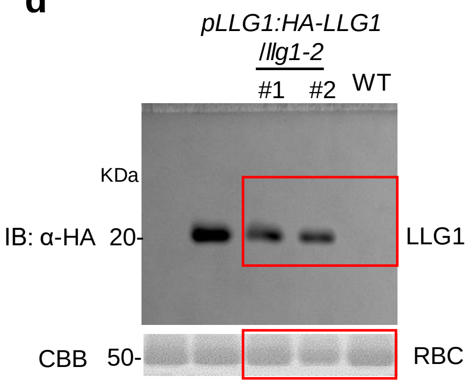

Source data Figure 5

h

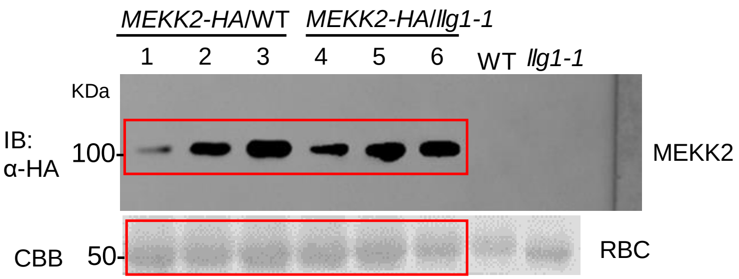

Source data Figure 6

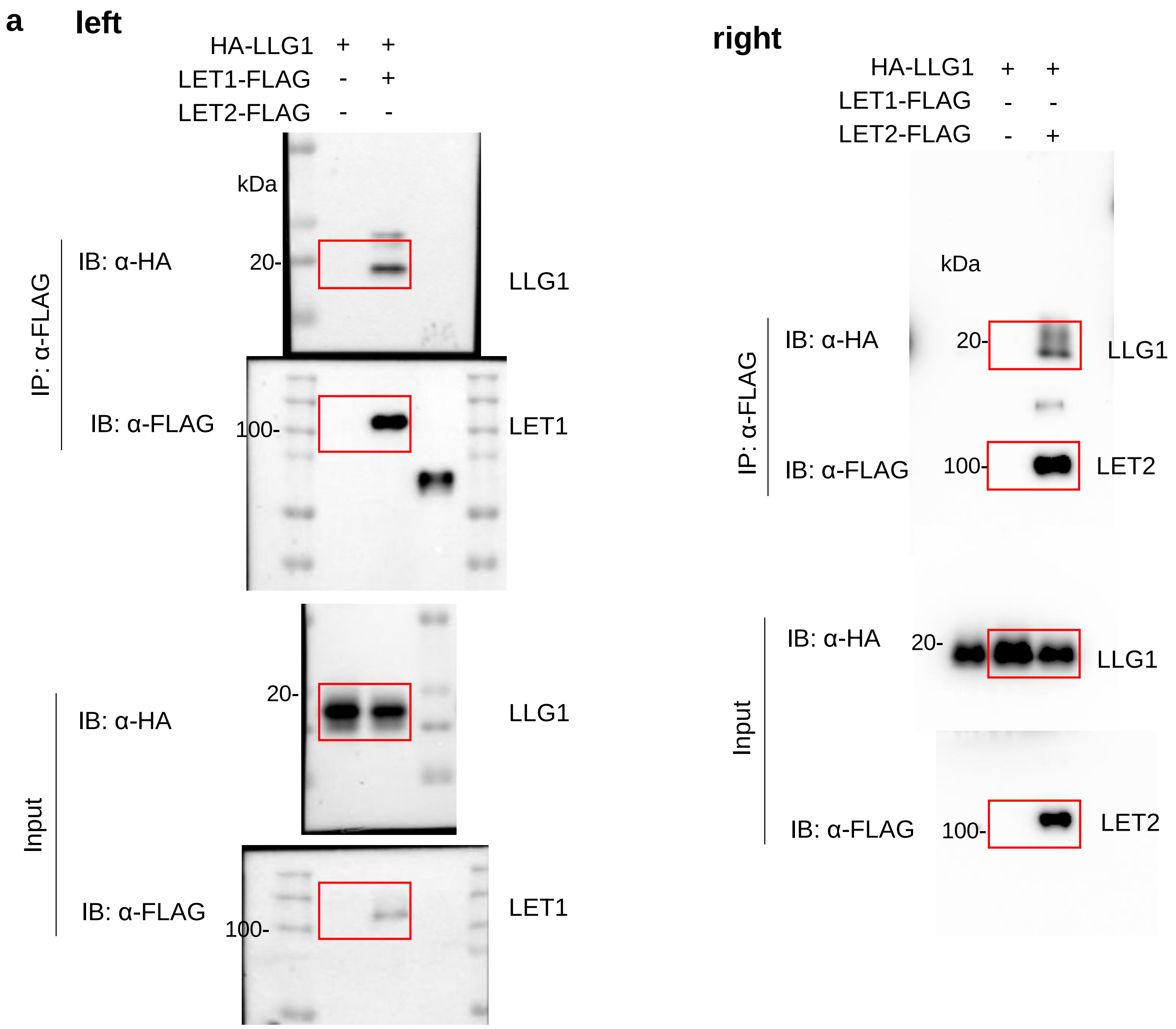

Source data Figure 7

b

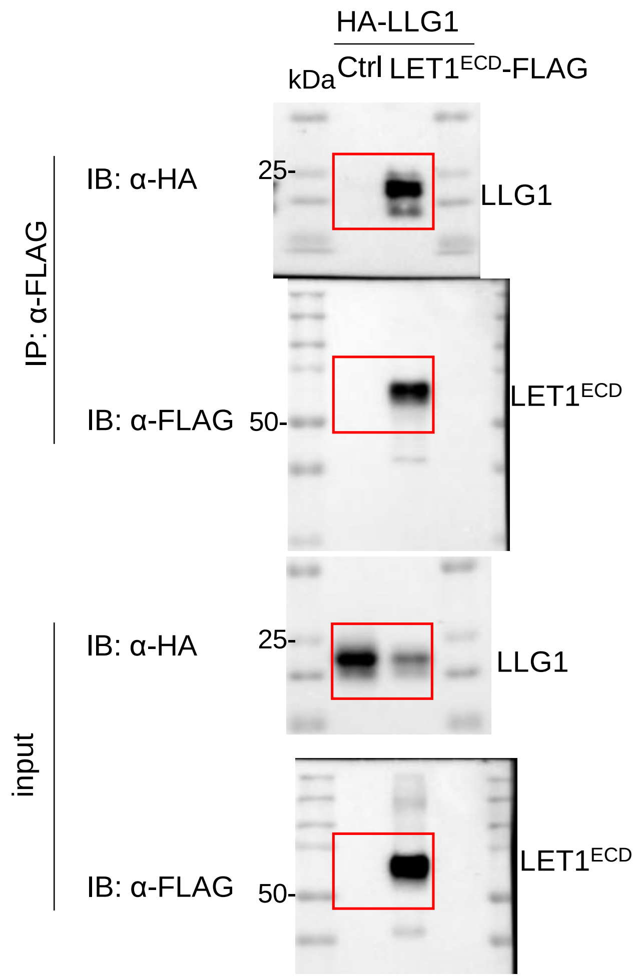

Source data Figure 7

c

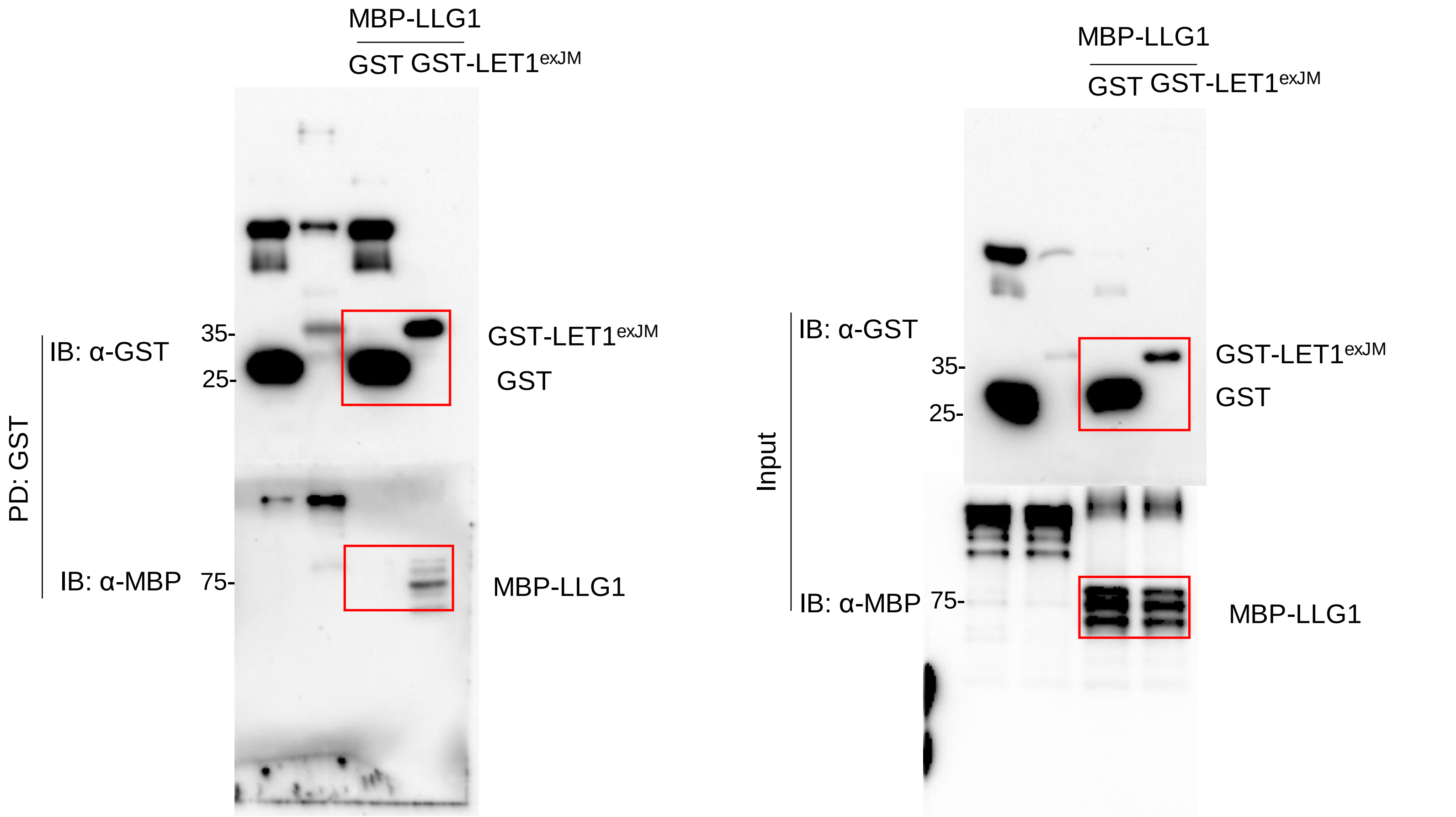
MBP-LLG1
GST GST-LET1exJM
PD: GST
IB: α-GST
35-
25-
GST-LET1exJM
GST
IB: α-MBP
75-
MBP-LLG1
Input
IB: α-GST
35-
25-
GST-LET1exJM
GST
IB: α-MBP
75-
MBP-LLG1

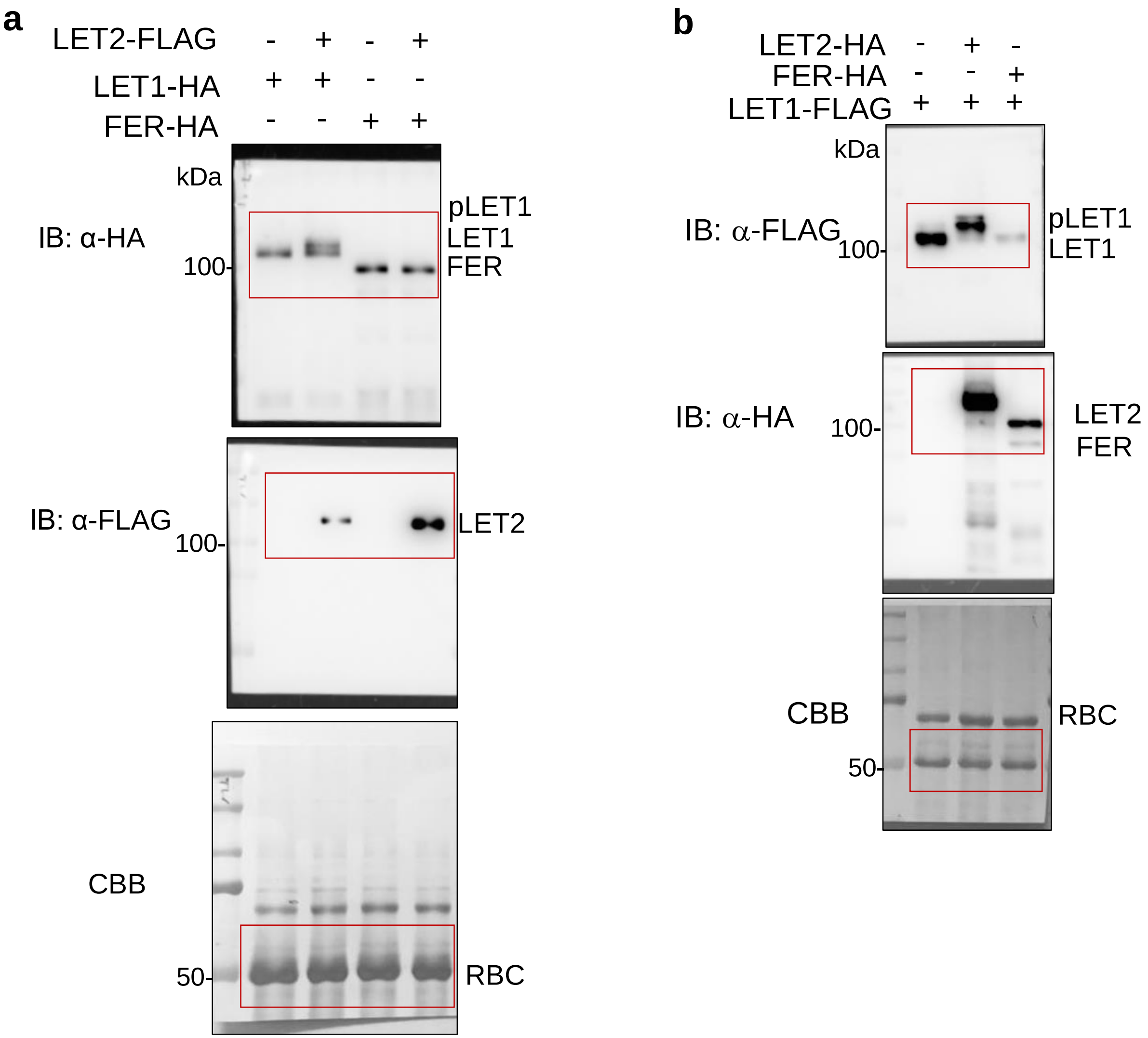

Source data Supplementary Figure 3

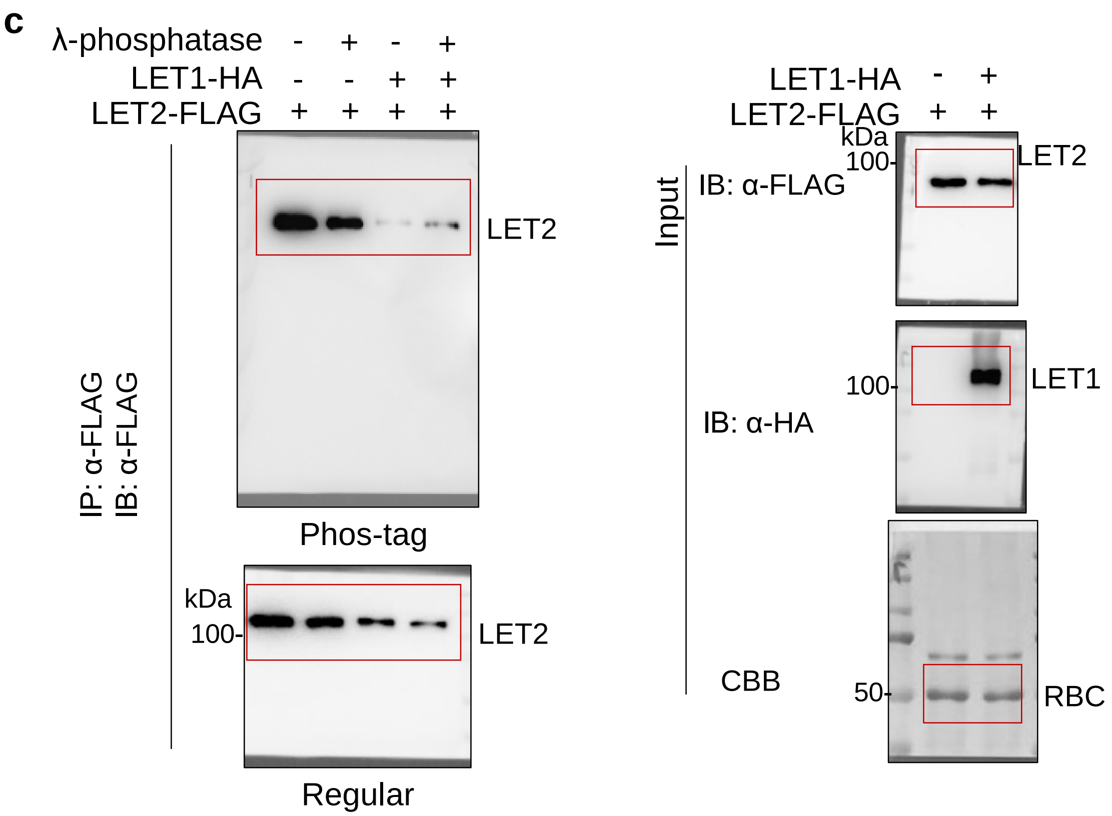

Source data Supplementary Figure S3

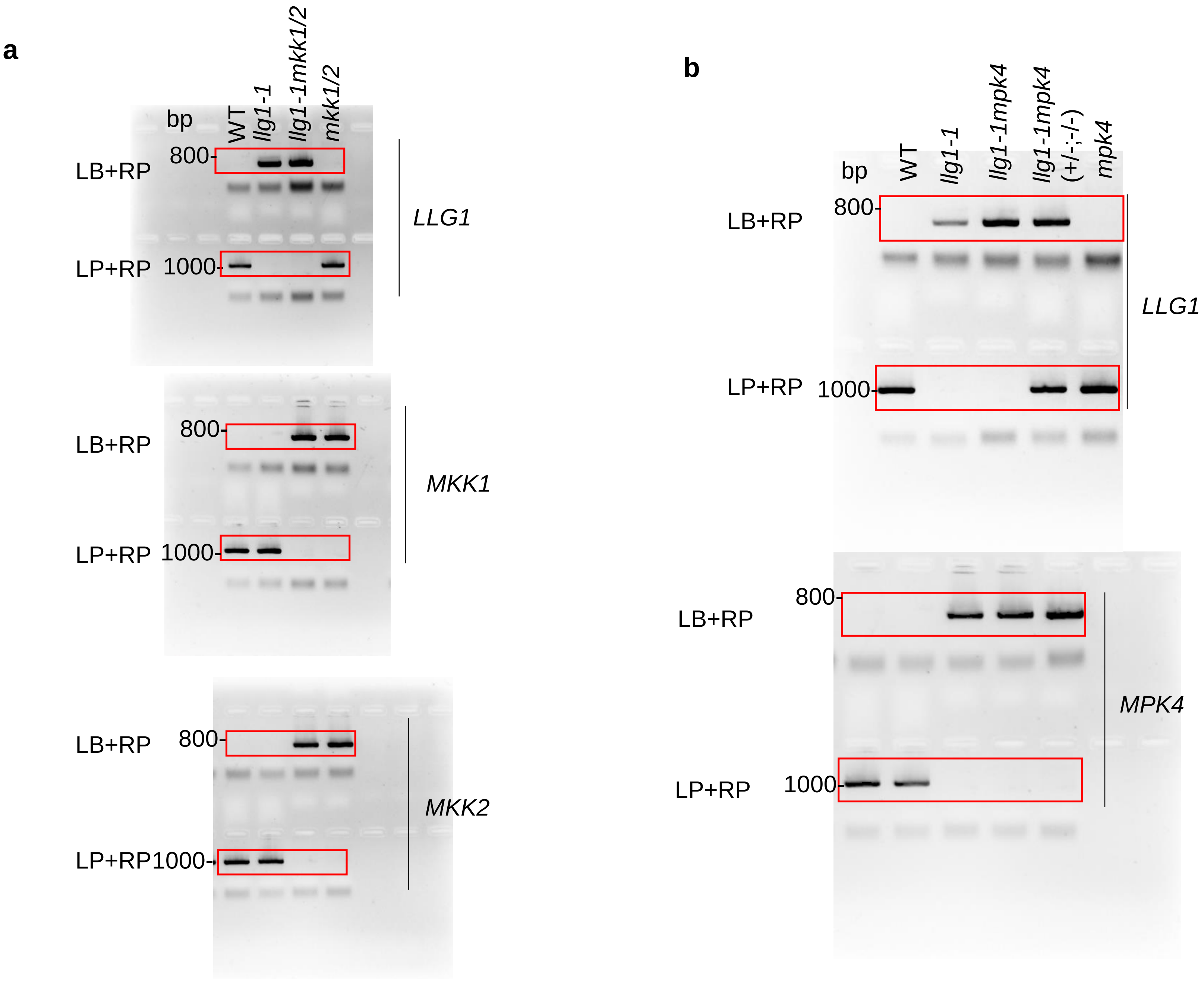

Source data Supplementary Figure S6

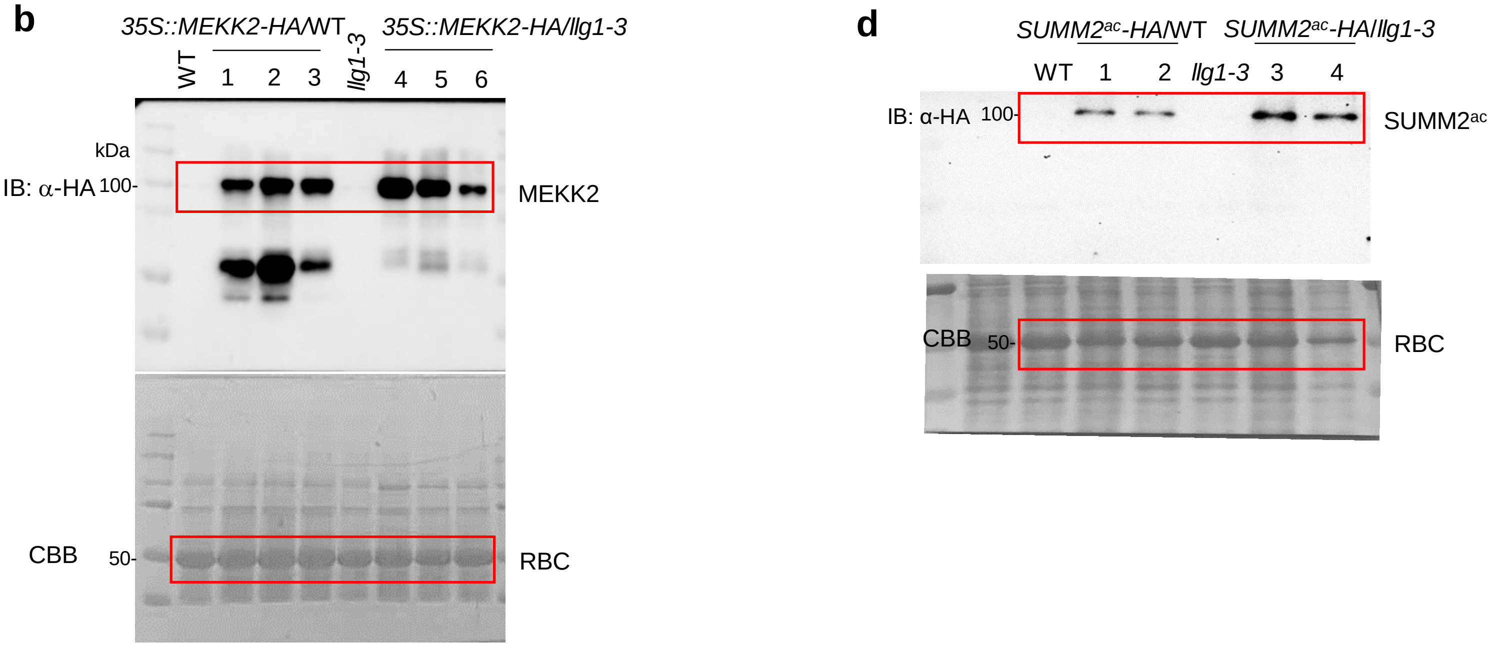

Source data Supplementary Figure S7
